# Supplementary figures and images for: Adipokine levels and their association with clinical disease severity in patients with dengue
Source: PLoS Negl Trop Dis. 2023 Sep 7;17(9):e0011613. doi: 10.1371/journal.pntd.0011613 (PMC10508597; doi:10.1371/journal.pntd.0011613)

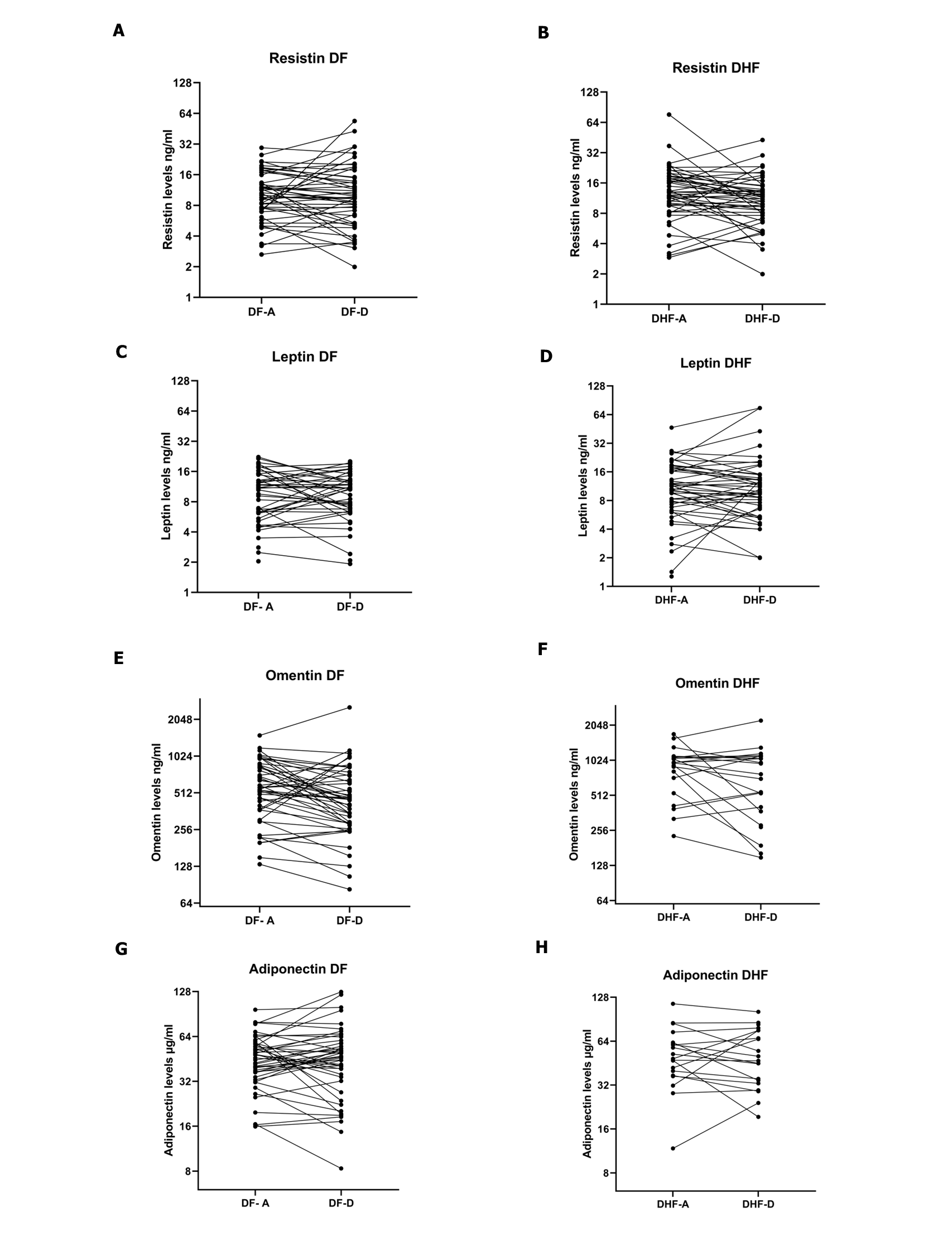

Supplement: S1 Fig — Resistin, omentin, leptin, and adiponectin levels were measured in patients with DF (n = 49) and DHF (n = 22) during the febrile and recovery phases, using a quantitative ELISA. The Wilcoxon matched pair singed-rank test was used to compare the levels of adipokines of the febrile (A sample) and the recovery phases (D sample) of patients with DF and DHF. The Mann-Whitney U test (two tailed) was used to calculate the differences in the means in the DF and DHF cohorts. The error bars indicate the median and the interquartile ranges. (TIF) [file pntd.0011613.s001.tif]
