# Supplementary material for: Adipokine levels and their association with clinical disease severity in patients with dengue
Source: PLoS Negl Trop Dis. 2023 Sep 7;17(9):e0011613. doi: 10.1371/journal.pntd.0011613 (PMC10508597; doi:10.1371/journal.pntd.0011613)
Supplement: Attachment — Submitted filename: Reviwer Comments - Final.docx [file pntd.0011613.s003.docx]

14^th^ May 2023

Dr. Benjamin L. Makepeace

Academic Editor

PLOS Neglected Tropical Diseases

Dear Dr. Makepeace,

**Adipokine levels and their association with clinical disease severity in patients with dengue**

We would like to express our gratitude to the reviewer for taking the time to thoroughly assess our manuscript and for providing valuable feedback. We have addressed all the comments raised and incorporated the necessary changes into the revised version of the manuscript, which is highlighted in the "Revised Manuscript with Track Changes" document.

**REVIEWER 01**

**Methods**

1. **Kuruppu et al have measured serum adipokines, a group of hormones released by fat cells, in patients early on in the clinical course of dengue infection, and then later in recovery. There has been very little work in this area and I believe this is the first report of this kind. A test to predict who will go on to develop severe disease would be very welcome, and although this paper mainly addresses adults these are still potentially helpful data, with some caveats.**

**The work is high quality and is written up well. The main deficiency is in the relatively small sample size. With some modifications, the work can be recommended for publication.**

Response: We thank the reviewer for carefully going through our manuscript and for all the comments, which we have addressed in the revised version.

**General points: One issue which is common in papers like this is there is no attempt at a sample size calculation, nor a post hoc attempt to describe what sort of difference could have been reliably detected using the number of patients with samples available. Sometimes it can be challenging in these settings to recruit adequate patient numbers, not having a pre-specified target does not invalidate the work. Nevertheless, it would be helpful for the reader to be able to assess how much could be due to chance, and if they were to repeat this work, what sort of sample they should be aiming for.**

**In terms of the specific review points, some general revisions such as stating the hypothesis tested more clearly, etc, could be better.**

Response: We thank the reviewer for your valuable feedback on the paper. We appreciate your suggestions regarding sample size calculations and the general hypothesis. Regarding the sample size calculation, we agree that this is an important aspect of any study and in this study, our aim was to explore if there were any changes in adipokine levels in patients with varying severity of illness. In such an event, if we calculate the sample size as assuming that the proportion of population having a difference in 50% (the default value), at a confidence interval of 95%, and a margin of error of 5%, the sample size would be 385. As the reviewer mentioned, as this is the first study, investigating adipokines in patients with acute dengue, and as we sampled at two time points to understand the changes in adipokine levels in the febrile and the critical phases of dengue, we only recruited 71 patients for this initial study. As we have observed some differences in adipokines in patients with different severity at different phases, we plan to investigate the mechanisms and if these can be used as biomarkers in a larger population of 385 patients.

As the reviewer has suggested, we have included a more details description of the hypothesis in the revised version of the manuscript.

**Conclusions**

1. **Line 335 – “adipokine IS likely to reduce” Also this statement is overstated – there was no difference in adiponectin levels with disease severity. The relationship is only with another marker. The clinical relevance of this is still to be shown – a larger study may be needed. Suggest this is revised to be more circumspect and to leave this as a hypothesis for future study.**

**The same is true of IFN beta in the discussion, as above for adipokines. This should be more cautiously worded. Having a better idea of the power of the study would also help here, including with corrections for multiple measurements (I appreciate this may not be straightforward due to the co-linear nature of some of the variables).**

**There are many interesting points in this article, which should be published. But the above points should be revised first.**

Response: We thank the reviewer for this comment. As very correctly mentioned by the reviewer, we only saw significant differences with omentin and used the term adipokines, as omentin is also an adipokine. We have revised this and also highlighted the need for a larger study in the discussion of the revised version of the manuscript. Again, with IFN beta, what we saw was a tend and we completely agree that a larger study is required, with correction for multiple measurements, to find out the true relevance of these findings. We have included this in the discussion.

**Editorial and Data Presentation Modifications**

1. **Line 75-76 – whilst the point about co-morbidities is well made, it would be more generalizable to say that severe disease is commonly seen in children, who usually don’t have such co-morbidities, but that these co-morbidities are highly relevant in adults, who can still develop severe disease.**

Response: Thank you for your valuable comment. We agree with the reviewer that many children who develop severe dengue do not have comorbidities. However, in many Asian and Latin American countries, dengue is predominantly seen in adults, and the presence of comorbidities are known risk factors for severe disease in adults, as very correctly mentioned by the reviewer. We have amended this in the revised version of the manuscript.

2. **Line 79 – “infected with DENV recover” (no need for “the”, and recover not recovery)**

Response: Thank you for your feedback, and we have made necessary improvements to our research and manuscript based on your suggestions.

3. **Line 83 – 91 Re COVID-19 and influenza: - true, but not really relevant or needed (eg also true in HIV, TB, sepsis, and many others etc). Better to point out that these co-morbidities are associated with worse outcomes from many infections as well as dengue. this isn’t a controversial statement and shouldn’t really need discussion of specific diseases to back it up. I see that later some of the inspiration comes from the COVID-19 literature – these points are well made but the way the comparison is introduced feels somewhat artificial.**

Response: Thank you for your feedback. We apologize for the comparison between COVID-19, influenza, and dengue. Our intention was to provide examples of infectious diseases where the association between comorbidities and worse outcomes has been well documented in the literature and where the mechanisms underlying these associations have been studied to some extent. However, we acknowledge that this can be stated clearly. Therefore, we have revise our wording to reflect the broader association between comorbidities and worse outcomes in infectious diseases without focusing specifically on COVID-19, influenza, or dengue. Thank you again for your feedback. We have made necessary improvements to our research and manuscript based on your suggestions.

4. **Line 134 – “fluid leakage WAS assessed”**

Response: Thank you for your feedback, and we have made necessary improvements to the manuscript based on your suggestions.

5. **Line 151 DEN or DENV?**

Response: Thank you for your feedback and for bringing this to our attention. We have made necessary improvements to our manuscript based on your suggestions.

6. **Line 172/3 – I don’t believe that space is particularly limited in PLOS NTD – I would strongly suggest that suppl table 1 is moved into the main text. This would be important information and with PLOS journals the supplements are always individual files, each of which have to downloaded – which is a pain. (Take note PLOS editorial teams!!)**

Response: We thank the reviewer for this comment and we have moved the supplementary figure into the main manuscript as suggested.

7. **Line 199-200 – how does recovery get called “D??” Why not just call this acute and recovery, or A and Rec if you really need it to be short? (I think you could fit in DHF with acute or recovery below.)**

We appreciate your attention on the details and helpful feedback. We have mentioned the recovery as “D” to indicate that it was collected on the date of discharge.

8. **What correction for multiple comparisons has been conducted for Fig 1?**

Response: In figure 1, we analyzed each adipokine during the early phase and recovery and we did not compare each adipokine with each other and we were not trying to find if any of these could be used as biomarkers in this study. Therefore, multiple comparisons were not done.

9. **There’s one outlier in Fig 2 for resistin levels – what happens when you take this sample out? Does any of the effect depend on this one sample? It would be much clearer if the r and p values from the Spearman tests were indicated on each panel in Fig 2 – please annotate them with this. (And indeed for all figs with this analysis.)**

Response: the statistical results do not change by removing the outlier. The r and p values for the Spearmans correlation has been included in the revised figure and not the figure legend as suggested by the reviewer.

10. **Line 224 – “As adiponectin/leptin ratio has BEEN shown to be a reliable marker of adipose tissue dysfunction” – Been missing. Also this needs a reference – it is I presume not referring to the present work.**

Response: We apologize for the missing reference and we have included references in the revised version.

11. **Line 321 – “infected by DENV” (no “the”)**

Thank you for your feedback and bringing this to our attention, we have made necessary improvements to our manuscript based on your suggestions.

**Reviewer 2**

**Methods**

**The work by Kuruppu et al. aims to explore the association between adipokine expression and dengue severity. The team enrolled 71 subjects, 49 with diagnosed with dengue fever and 22 with dengue hemorrhagic fever. A number if adipokines (resistin, omentin, leptin, and adiponectin) are measured in patients at admission and discharge. The research team also measured key liver enzymes, viral load, NS1 and the anti-inflammatory cytokine, IFNb. While the team has generated a nice dataset there are a few limitations.**

**1. Given the major focus is on adipokines, the paper would benefit from measuring plasma lipid biomarkers. This is especially relevant since the Sri Lankan diet consists of saturated oils such as coconut oil and therefore preexisting lipid levels may confound the interpretation of the adipokine expression data. If sample does not exist to do anymore plasma analysis, could the BMI of each patient be calculated? The BMI may act as a surrogate for lipid profile.**

**Response:** We thank the reviewer for the valuable feedback on the manuscript. We appreciate your suggestion to measure plasma lipid biomarkers to complement the analysis of adipokine expression data. However, many studies have shown that the plasma lipids are very different and significantly lower in acute dengue, when compared to the healthy states (please see references below). Although the BMI would have given an indication of the extent of obesity, as the patients were acutely ill, and again many studies have shown conflicting results between the association of BMI and the lipid profile especially in patients who have a higher muscle mass and in different ethnicities. Due to this conflict in the existing data between BMI and lipid profiles, we did not use this as a surrogate marker (please see references below).

Studies that show serum total cholesterol and other cholesterol components being lower in acute dengue:

- Biswas et al, Lower Low-Density Lipoprotein Cholesterol Levels Are Associated with Severe Dengue Outcome, 2015: PMID: 26334914
- Lima et al, Serum lipid profile as a predictor of dengue severity: A systematic review and meta-analysis, 2019, PMID: 31172625
- Van Gorp, Changes in the Plasma Lipid Profile as a Potential Predictor of Clinical Outcome in Dengue Hemorrhagic Fever, 2002, PMID: 11915007
- Suvarna et al, Serum lipid profile: a predictor of clinical outcome in dengue infection, 2009, PMID: 19309479

**Studies showing varying correlation between BMI and lipid profiles**

[Body mass index : considerations for practitioners (cdc.gov)](https://stacks.cdc.gov/view/cdc/25368)

Different correlation of body mass index with body fatness and obesity-related biomarker according to age, sex and race-ethnicity: PMID: 36859451

Physiology. The health risk of obesity--better metrics imperative: PMID: 23970691

Body Mass Index: Obesity, BMI, and Health: A Critical Review: PMID: 27340299

**2. The study team should consider providing more demographic information of the dengue cohort. For example, the paper would benefit from a table that has not only age, gender (which is noted in the manuscript as text) but also comorbidities such as percent with diabetes, cardiovascular disease, COVID-19 etc. This information can then be used to reinterpret data from Figure 1.**

Response: Thank you for your valuable feedback on the manuscript. We appreciate your suggestion to provide more demographic information about the dengue cohort, including comorbidities such as diabetes, cardiovascular disease, and COVID-19, to better interpret the data in Figure 1.

We agree that this additional information would be useful for understanding the characteristics of the cohort and their potential impact on the findings. In the dengue cohort, we had a very limited number of patients (9/71) with comorbidities, all of whom were controlled with routine medication. We have included this information in the revised version of the manuscript.

**3. There is little to no information about the healthy cohort. Are these individuals age-matched, gender-matched, diet-matched or clinically matched for other underlying ailments? This is relevant given that adipokine levels can change as a result of all these variables. In addition, the median of the healthy group look similar to the infected groups at admission, which suggest that what the authors are seeing maybe due to other factors. The only adipokine that demonstrates marked differences between the healthy and infected groups is omentin.**

Response: We thank the reviewer for this comment and agree that if not age or clinically matched that it may affect the results. The healthy cohort was recruited from the same geographical region as the dengue cohort, and all healthy individuals were matched for age, gender and were consuming a Sri Lankan diet. We apologize for not including this information and we have done so in the revised version of the manuscript.

**4. The Spearman correlations albeit significant are quite weak with a Spearman’s coefficient which ranges from 0.3-0.4. Therefore it should be noted in the manuscript that these are fairly weak.**

Response: We agree with the reviewers comment and made this amendment in the revised version of the manuscript.

**Results**

1. **The authors should consider adding more information to Table 1 such as age, gender, and prior clinical histories.**

Response: Thank you for your helpful comment and we have included this data in Table 1 the age and gender of the participants.

2. **The authors should add a column for the p-values comparing the adipokine levels at admission and discharge. This makes it much easier for the reader.**

Response: We thank the reviewer for this comment. Based on how the data is presented in table 1, as it is difficult to add p values between admission and discharge between thr 2 groups, we have included this information as a supplementary table 1, in the revised version.

3. **The authors may want to consider a different way to visualize the data in Figure 1. For example rather than dot plots maybe spaghetti plots to see the change of each cytokine for a given individual between admission and discharge.**

Response: We thank the reviewer for this suggestion. We have done this in the revised version of the manuscript and presented the data as supplementary figure 1. As spaghetti plots show how data varies over a period, all the data points are connected and therefore, the graph would not allow the display of data of those with DF and DHF in the same graph as these will be connected. As we thought it would be appropriate to display the data points of DF and DHF in the same graph along with the healthy, we have included the suggested figure as a supplementary figure 1.

4. **The authors should also consider presenting Figure 2 for the DF and DHF groups separately.**

Response: We thank the reviewer for this suggestion, and we have done this in the revised manuscript.

5. **The authors should consider presenting a figure of the adipokine levels for those patients from the dengue fever group who progressed on to get DHF. Is there a difference in particular adipokines? Given that data is presented for the entire cohort it would be important to see if those patients that went on to get DHF expressed more of one marker over the other.**

Response: We thank the reviewer for this question and we apologize for the lack of clarity. All patients for the study we recruited (time of taking sample A), before they had developed any features of vascular leakage/DHF. Therefore, those who we have classified as DHF, had did not have any features of DHF when sample A was taken (DHFA). Those we classified as DF remained as having DF and never developed any features of DHF. We have clarified this in the revised version of the manuscript.

6. **The paper would have benefited from an analysis of different cellular subsets. In the cohort, how dysregulated was the T cell repertoire? Given that M1 macrophages are induced by adipokines, did the cohort express these cells. If the study team has access to the cells I would strongly suggest this piece of work.**

Response: We agree with the reviewer that this indeed would have provided a better understanding of the role of adipokines in disease pathogenesis. However, studying the T cell repertoires and monocyte responses was beyond the scope of this study. With this data, we plan to study T cell responses and monocyte responses in patients with varying severity of acute dengue in our future studies.

**Conclusions**

1. **The paper by Kuruppu et al. provides a descriptive analysis of the association between adipokines and dengue severity. However, the authors may be overstating some of their conclusions given the weak correlations seen in Figure 2. Therefore, the authors should consider tempering their conclusions to be more suggestive. Furthermore, the authors should explain the rationale to measure IFNbeta and to use NS1. The study team measures NS1, provides the results but there is no mention in the conclusions. If NS1 was used to differentiate between the different dengue serotypes, this should be mentioned.**

**The topic is interesting and worth publishing given the importance of understanding how dengue immunity can be shaped by factors such as diet, obesity etc. The research team performs a nice piece of work but several modifications are required before publication.**

Response: We would like to thank the reviewer for their valuable comments on our study. We have revised our conclusions as suggested by the reviewer and explained our basis for using IFN beta and NS1 in our studies. We did not use NS1 to differentiate between dengue serotypes, as we had done this using real-time PCR. We used NS1 as it has been shown to associate with clinical disease severity and IFN beta as it a potent antiviral cytokine. We have explained giving references in the revised version of the manuscript.

**Editorial and Data Presentation Modifications**

1. **line 79 - remove "the" and change "recovery" to "recover"**

Thank you for your feedback and bringing this to our attention, we have made the necessary corrections to our manuscript based on your suggestions.

2. **page 4, line 81 - consider rephrasing this sentence to "those who develop DHF have an altered and dysfunctional immune response characterized by ..."**

Thank you for your feedback and bringing this to our attention, we have made the necessary improvements to our manuscript based on your suggestions.

3. **page 4, line 85 - remove "as seen with" and replace with "and"**

Thank you for your feedback and bringing this to our attention, we have made the necessary improvements to our manuscript based on your suggestions.

**Summary and General Comments**

**This manuscript would benefit from the following experiments:**

**1) Measure plasma lipid markers**

Response: Thank you for your feedback on our paper. As shown in numerous references, the plasma lipids are significantly altered during dengue and therefore, will not reflect the actual state of the lipid profiles in the patients in health. Therefore, we did not measure these as it would not provide any additional information in a diseases state.

**2) If cells are available for the different time points, FACS assays to explore T cell subsets and M1 macrophages would be enlightening.**

Response: We thank the reviewer for this suggestion, but we did not collect or store PBMCs from these patients.

**3) The omentin data is intriguing given that this adipokine is the only one that was higher in the dengue-infected groups as compared to the healthy group. The team should consider measuring IL-25 in the study groups.**

Response: Thank you for your valuable feedback regarding our manuscript. We appreciate your suggestion to measure IL-25 levels in the study groups, given the intriguing data on omentin levels in the dengue-infected groups. Unfortunately, we do not have any serum left from most of these patients as numerous assays were done in this study.

We agree that measuring IL-25 levels could provide additional insights into the immune response and dysregulation of adipokine levels associated with the disease. We will consider measuring IL-25 levels in future studies to further explore the relationship between adipokines and immune responses in the context of this disease.

**4) The authors should consider re-analyzing the data by clinical history to assess whether new observations can be made. In examining the cohort as a bulk group the authors may be missing out on some interesting data.**

Response: We apologize but this suggestion is not clear to us. Clinical data was recorded from all patients each day from the day they were admitted, until they were discharged. Patients were classified as having DF or DHF based on this data. We are not clear about analyzing possible new observations.

**Reviewer 3**

**Methods**

1. **Can the authors describe the clinical features suggestive of acute dengue that were the inclusion criteria for recruitment? What are the exclusion criteria?**

Response: We thank the reviewer for the comment. In this study, the inclusion criteria were clinical features suggestive of acute dengue, such as fever, rash, myalgia, arthralgia, bleeding, and other laboratory investigations. The exclusion criteria were history or clinical evidence of other acute infections (urinary tract infection, cellulitis, or tonsillitis), chronic liver and kidney disease. We have included this information in the revised version of the manuscript.

2. **Please mention, the total number of patients that were recruited based on the clinical features, prior to the inclusion of 71 patients for the study.**

Response: We thank the reviewer for this comment. Based on the study, the total number of patients recruited based on clinical features prior to the inclusion of the 71 patients for the study was 140. We have included this information in the revised version of the manuscript.

3. **It is not clear whether this was a retrospective/prospective study or did the authors work on archived blood specimens from an earlier study.**

Response: We apologize for the lack of clarity. This was a prospective study based on the information provided in this study. Blood specimens were collected at two time points in each patient: the acute phase and recovery phase of the study period. We have included this information in the revised version of the manuscript.

4. **Was there enough statistical power to test the study's hypotheses?**

Response: The main aim of this study was to find out adipokines could be playing a role in dengue disease pathogenesis. We found many adipokines were elevated in patients with acute dengue compared to healthy individuals and that some adipokines were significantly elevated in patients who progressed to develop DHF. As the main aim of the study was not to see if such adipokines could be used as predictive markers, but to understand if there were changes between the healthy state and in those with acute dengue, the sample size appears to be adequate to test the hypothesis.

5. **Please describe the study period/patient recruitment period?**

Response: We thank the reviewer for this question. The patient recruitment and sample collection period was from November 2021 to August 2022. During this period, we recruited patients who met the inclusion criteria and collected blood specimens at two time points in each patient: the acute phase and recovery phase of the study period.

6. **Can the authors include the number of days since disease onset before the DF patients progressed to develop DHF.**

Response: We apologize for the lack of clarity. As mentioned in the methods, the number of days since disease onset before DF patients progressed to develop DHF was less than four days.

7. **Blood specimen collection from healthy individuals for measurement of adipokines and INF-beta levels must be described.**

Response: Thank you for this question and we apologize for not adequately elaborating on this in our manuscript. We recruited 18 healthy individuals for this purpose, and all individuals were selected according to age, gender, and diet matched criteria. Blood specimens were processed according to established protocols, and adipokine and INFβ levels were measured using commercially available ELISA kits. We have included this information in the manuscript to make it more transparent.

**Results**

1. **Table 1, the abbreviations (eg. IQR, A, D) should be described.**

Response: We thank the reviewer for this, we have made the necessary improvements to our manuscript based on your suggestions.

2. **Table 1, the empty boxes should be labelled 'not applicable' or 'not performed'.**

Response: We thank the reviewer for this, we have made the necessary improvements to our manuscript based on your suggestions.

3. **The viral loads seemed low to me. It is a little odd to find acute viral loads at these levels.**

Responses: We thank the reviewer for this question. There have been wide variations with viral loads in many studies, in patients infected with different serotypes and in those with primary and secondary dengue (Vuong et al, 2021). Viral loads have shown to be lower in those infected with DENV2 than with other serotypes. As the majority of patients recruited in our study, were infected with DENV-2, there could be a trend towards lower viral loads than in studies describing infection with DENV1 and DENV3. However, many studies have described a wide variation in viral loads in patients.

4. **Is there any explanation why leptin levels are relatively similar between Healthy, DF and DHF?**

Response: We thank the reviewer for this observation. What our data appears to show that unlike other adipokines there was no change in leptin levels in patients with acute dengue compared to the healthy state. Therefore, although many other adipokines and inflammatory mediators change during acute dengue, as the leptin levels remained more or less the same as in the healthy state, leptin (and leptin pathways) are unlikely to be involved in dengue disease pathogenesis.

**Conclusions**

1. **The authors showed the potential of adipokines for use as prognostic markers for dengue. However, it must be noted that this study was performed on a comparatively low number of study participants. The present study is best described as a preliminary study showing a variety of adipokines with different expression levels between patients with DF and DHF. A larger cohort is required to corroborate the findings from this study.**

Response: We thank the reviewer for this observation and the apologize for giving the impression that adipokines could be used as prognostic markers based on this study. We completely agree with the reviewer that our study was performed with a relatively small sample size as the aim of the study was not to investigate if adipokines could be used as a prognostic marker. Since our data shows that adipokines are likely to have some role in disease pathogenesis, follow up studies are required to find out the exact mechanisms and studies on larger cohorts are required to find out if it can be used as a prognostic marker. We have described these in the revised version of our manuscript and have suggested that future studies should focus on larger cohorts and investigate the potential of adipokines as prognostic markers in predicting disease severity and outcomes in dengue patients.

**Summary and General Comments**

1. **This is an interesting study looking at the different adipokine levels between DF and DHF patients. It would be even interesting to see the different adipokine levels between patients infected with different dengue serotypes. My major concern is on the number of study participants, which in my opinion, has to be larger; especially in Sri Lanka where dengue is endemic. There are several typos too (Line 79-recovery, Table 1-0.34 to 0.07 to 4 & 0.3 to 0.17 to 1).**

Response: Thank you for your valuable feedback. We agree with your comment that our study was performed with a relatively small sample size and can be considered as a preliminary study. While we believe that our findings are important and contribute to the current understanding of the role of adipokines in dengue infection, we acknowledge that further studies with larger cohorts are needed to confirm and expand our findings. We have highlighted this in the summary section of our manuscript and have suggested that future studies should focus on larger cohorts and investigate the potential of adipokines as prognostic markers in predicting disease severity and outcomes in dengue patients. We have made the necessary improvements to our manuscript based on your suggestions.

Thank you for considering our manuscript with PLOS Neglected Tropical Diseases.

Yours Sincerely,

Prof. Neelika Malavige
