# Supplementary material for: Adipokine levels and their association with clinical disease severity in patients with dengue
Source: PLoS Negl Trop Dis. 2023 Sep 7;17(9):e0011613. doi: 10.1371/journal.pntd.0011613 (PMC10508597; doi:10.1371/journal.pntd.0011613)
Supplement: Attachment — Submitted filename: Reviwer Comments 01.08.2023.docx [file pntd.0011613.s004.docx]

1^st^ August 2023

Dr. Benjamin L. Makepeace

Academic Editor

PLOS Neglected Tropical Diseases

Dear Dr. Makepeace,

**Adipokine levels and their association with clinical disease severity in patients with dengue**

We would like to express our gratitude to the reviewer for taking the time to thoroughly assess our manuscript and for providing valuable feedback. We have addressed all the comments raised and incorporated the necessary changes into the revised version of the manuscript, which is highlighted in the "Revised Manuscript" document.

**Reviewer 1**

**1. I’m grateful to the authors for their time in responding to my points about the manuscript. I remain of the view that these data are helpful to have in the published literature. Unfortunately, I don’t think the authors have gone far enough yet in addressing the limitations of their study.**

**Overall, I find the response regarding the statistics to be unsatisfactory. There is not sufficient discussion about the limitations of the study in the discussion. There is a lot of discussion about the possible biology of adipokines in inflammation. But this discussion may not be so relevant if the findings cannot be replicated. I can’t quite follow the sample size calculation – to detect a 50% change in a marker can be done with far fewer people depending on what the variance of the data is, I don’t see this clearly addressed. The following need to be addressed in the manuscript for it to be recommended for publication: Sample size and maximum difference that could have been detected**

**Response:** We thank the reviewer for the insightful review of our manuscript. We aplogise for not including a section on statistical analysis, which we have included in the revised version of the manuscript. To address the potential heterogeneity of variances in our data, we conducted a test for equal variances using Levine's test based on absolute deviation from the mean. The results of this test indicated that there were no significant differences in the variances among the samples (p value > 0.05). This finding supports the robustness of our analysis and ensures the validity of our results. We have included these details in the revised manuscript.

**2. Specify whether multiple comparisons have been conducted or not**

**Response:** Thank you for your valuable feedback on our manuscript. We did not correct for multiple comparisons as the reviewer very correctly has mentioned that in this study, we wished to assess if any of the adipokines were elevated or reduced in patients with dengue and if there were differences with disease severity. We have included this in the revised manuscript.

**3. Section on limitations of the study which should specifically acknowledge these weaknesses and point out that these findings need to be replicated**

Response: We thank the reviewer for this suggestion. We have included this in line 268.

**4. Removal of most of the discussion about the biology of the markers, eg especially the comments about adipokines and IFN beta in the discussion (line 335 and below in the original manuscript) remain overstated – on further reflection I think these should be removed altogether.**

**Response:** We have revised the manuscript as suggested by the reviewer and have removed this part.

**5. Making the raw data available will help anyone interested in doing similar work.**

Thank you for your valuable feedback on our manuscript. We highly appreciate your suggestion regarding the availability of raw data. We fully support the idea of making the raw data available to anyone interested in conducting similar work or wishing to verify our findings. The data can be made accessible upon request to ensure that interested individuals have the opportunity to utilize the dataset for further research.

**6. Regarding point 7 – if D indicates discharge, the please use a clearer abbreviation such as “Disch.” Please relabel the axes of Fig 1 so that the reader can appreciate this more clearly – DF-A should be “Acute” and DF-D “Disch” – I suggest then putting the disease state underneath as a sub title. This will be a lot clearer.**

Response: Thank you for your valuable feedback on our manuscript and for suggesting clearer abbreviations for the disease state at the time the 2^nd^ sample was obtained. In response to your comment, we have reworded the abbreviation for the first sample (Day 3 or 4), which is now labeled as "A" to represent the "Acute" phase. Additionally, the second sample (Day 5 or 6) is now labeled as "B" to signify the "later stages of critical illness". We have also incorporated these changes in the figure legend to provide a clearer understanding to the reader. Furthermore, we have relabeled of Figure 1 as "Acute (A)" and "later stages of critical illness (B)" to ensure a more straightforward and concise representation of the disease states.

7. **“Unlike as seen with the virus, the NS1 antigen was not more likely (p=0.41) to be positive in those who had DHF (odds ratio 0.55, 95% CI 0.19 to 1.6).” – please remove “unlike as seen with the virus – as this was not significant either.**

Response: Thank you for your careful review of our manuscript and for providing constructive feedback. In response to your comment, we have made the necessary change in line 246 and removed the phrase "unlike as seen with the virus." We agree that this addition was not significant either and might have caused confusion. The revised sentence now reads as follows: "The NS1 antigen was not more likely (p=0.41) to be positive in those who had DHF (odds ratio 0.55, 95% CI 0.19 to 1.6)." We appreciate your attention to detail, and we believe that this change improves the clarity and accuracy of the statement in our manuscript.

**8. “between a defunct type I interferon response” – defective type I interferon**

Response: Thank you for your review of our manuscript. We appreciate your feedback. In response to your comment, we have made the necessary change to the phrase.

9. **“However, when analyzed separately, while there was no correlation of AST levels with serum resistin levels in patients with DF, serum resistin levels during the febrile phase were significantly correlated with AST levels (r=0.21, p=0.03, Figure 2F) in those with DHF, although this association was weak.” – suggest splitting this into two sentences**

Response: Thank you for your valuable feedback on our manuscript. We have carefully considered your suggestion and have split the sentence into two to improve clarity and readability.

10. **“possible due to small number (n=9) of individuals” – suggest replace with “but the study was limited by the small number of patients with metabolic disease (n=9).”**

Response: Thank you for your valuable feedback on our manuscript. We have considered your suggestion and have made the appropriate change in line 294.

Thank you for considering our manuscript with PLOS Neglected Diseases.

Yours Sincerely,

Prof. Neelika Malavige
