# Supplementary material for: Adipokine levels and their association with clinical disease severity in patients with dengue
Source: PLoS Negl Trop Dis. 2023 Sep 7;17(9):e0011613. doi: 10.1371/journal.pntd.0011613 (PMC10508597; doi:10.1371/journal.pntd.0011613)
Supplement: Attachment — Submitted filename: Editorial comments 15.08.2023.docx [file pntd.0011613.s005.docx]

15^th^ August 2023

Dr. Benjamin L. Makepeace

Academic Editor

PLOS Neglected Tropical Diseases

Dear Dr. Makepeace,

**Adipokine levels and their association with clinical disease severity in patients with dengue**

We would like to express our gratitude to the reviewer for taking the time to thoroughly assess our manuscript and for providing valuable feedback. We have addressed all the editorial comments below.

**From a scientific perspective, I am satisfied that you have revised the manuscript to the sufficient standard. However, note that it is a condition of publishing in PLoS journals that all non-sensitive raw data are made publicly available.**

**Response:** we are delighted that our manuscript was accepted. The raw data is uploaded in the revised version as supplementary raw data.

Thank you for considering our manuscript with PLOS Neglected Diseases.

Yours Sincerely,

Prof. Neelika Malavige
